# One-Step Sol–Gel-Fabricated CuZn Alloy Aerogel Enabled by Cu–Zn Bimetallic Synergy for Efficient Antibacterial and Anti-Biofilm Therapy

**DOI:** 10.3390/gels12090815

**Published:** 2026-09-06

**Authors:** Lin Teng, Zhiqiang Zhou, Changyuan Feng, Guoyuan Li, Weihao Men, Yun Cui, Shuo Liu, Libing Zhang

**Affiliations:** 1 School of Energy and Chemical Engineering, Tianjin Renai College, Tianjin 301636, China; teng_lin@tju.edu.cn (L.T.);; 2 Tianjin Key Laboratory of Molecular Optoelectronic Sciences, Department of Chemistry, School of Science, Tianjin University, Tianjin 300072, China

**Keywords:** alloy aerogels, antibacterial, anti-biofilm, reactive oxygen species, membrane potential

## Abstract

Copper nanoparticles possess broad-spectrum antibacterial activity, and aerogels with 3D interconnected porous networks can trap bacteria and sustain metal ion release to boost bactericidal effects. Zinc is another low-toxicity antibacterial metal, and the Cu–Zn combination is predicted to generate synergistic inhibition. Herein, monometallic Cu aerogel and CuZn alloy aerogel were fabricated by a one-step method, and comparative experiments were performed to verify whether Zn alloying improves the antibacterial performance of Cu aerogel. TEM and XRD suggest the probable formation of Cu–Zn substitutional solid solution; Zn addition refined nanoparticles and relieved particle aggregation. Quantitative viability tests, agar diffusion and biofilm inhibition assays proved that CuZn alloy aerogel exhibited superior bactericidal and anti-biofilm activity against *E. coli* and *S. aureus*. Mechanistic investigations revealed that the bimetallic alloy induced strain-dependent intracellular ROS accumulation and disrupted bacterial membrane potential to cause irreversible bacterial death. DC2.4 cell tests validated its good cytocompatibility, with cell viability over 70% at 100 ppm, the concentration delivering excellent antibacterial capacity. This work explores the combined antibacterial advantages of Cu-Zn bimetallic alloy aerogel and offers a facile strategy to fabricate biocompatible metal aerogels for biomedical antibacterial applications.

## 1. Introduction

Microbial infection has always been a severe threat to global public health and human safety. The inappropriate use and overuse of traditional antibiotics in clinical treatment and daily life have induced the widespread emergence of bacterial drug resistance, which greatly reduces the therapeutic effect of conventional antibiotics [1,2,3,4]. Consequently, it is urgent and essential to develop high-efficiency, safe and durable alternative antibacterial materials to replace traditional antibiotics [5,6,7].

With the rapid advancement of nanotechnology, nanomaterial-based antibacterial agents have achieved remarkable progress and emerged as promising alternatives to traditional antibiotics [8,9,10]. On the one hand, nanomaterials can serve as excellent carriers for the controlled delivery and sustained release of antibacterial active substances. On the other hand, some nanomaterials exert inherent bactericidal performance through multiple physical and chemical mechanisms, including destroying bacterial cell structure, regulating intracellular oxidative stress, and interfering with bacterial metabolic processes. Among diverse antibacterial nanomaterials, copper- and zinc-based nanomaterials have attracted extensive research attention due to their low biological toxicity, broad-spectrum bactericidal activity, stable chemical properties and low risk of inducing bacterial resistance, showing great application prospects in antibacterial and disinfection fields [11,12,13,14].

Aerogels are a class of emerging ultra-light three-dimensional porous nanomaterials with high specific surface area, high porosity and interconnected network structure [15,16,17]. Benefiting from their unique structural characteristics, aerogels can provide abundant adhesion sites for bacteria and load antibacterial active components uniformly, which effectively improves the contact efficiency between materials and bacteria. Different from dense traditional materials, the porous structure of aerogels may facilitate the release of antibacterial metal components, which is promising for achieving long-lasting antibacterial performance. Such superior structural and functional properties make aerogels ideal substrate materials for constructing high-performance antibacterial composites [18,19,20].

Recently, alloy aerogels have been explored for antibacterial applications, demonstrating great potential in solving bacterial infection problems. For instance, noble metal-based Pd alloy aerogels integrate unique photothermal performance and multi-enzyme-like catalytic activity, realizing synergistic photothermal-chemodynamic antibacterial therapy toward drug-resistant bacteria [21]. Transition-metal Mn-Ce-Zn ternary alloy aerogels achieve dual functions of environmental purification and broad-spectrum bacterial inhibition via ROS generation and metal ion release [22]. Furthermore, recent review works have systematically summarized the structure–activity relationships and synergistic mechanisms of multimetallic alloy antibacterial materials, and pointed out the existing bottlenecks including complex preparation, unstable performance and insufficient mechanism exploration, which further inspire the rational design of novel alloy aerogel antibacterial platforms [23].

Copper nanoparticles are widely recognized as efficient broad-spectrum antibacterial agents [24,25,26]. Aerogels possess interconnected three-dimensional porous networks and large specific surface areas, which can trap bacteria and realize sustained release of metal ions, thus promising to enhance the inherent antibacterial performance of copper. Meanwhile, zinc is another low-toxicity metal with inherent bactericidal activity, and the combination of Cu and Zn is expected to produce synergistic antibacterial effects. Inspired by these facts, we designed and fabricated CuZn alloy aerogel in this work, with monometallic Cu aerogel set as the control sample. A series of structural characterization, antibacterial and anti-biofilm tests, as well as mechanistic exploration and cytocompatibility evaluation, were carried out to systematically compare their performance differences and reveal the antibacterial mechanisms originating from bimetallic Cu-Zn combination.

## 2. Results and Discussion

### 2.1. Characterization of Cu and CuZn Aerogels

**Figure 1:**
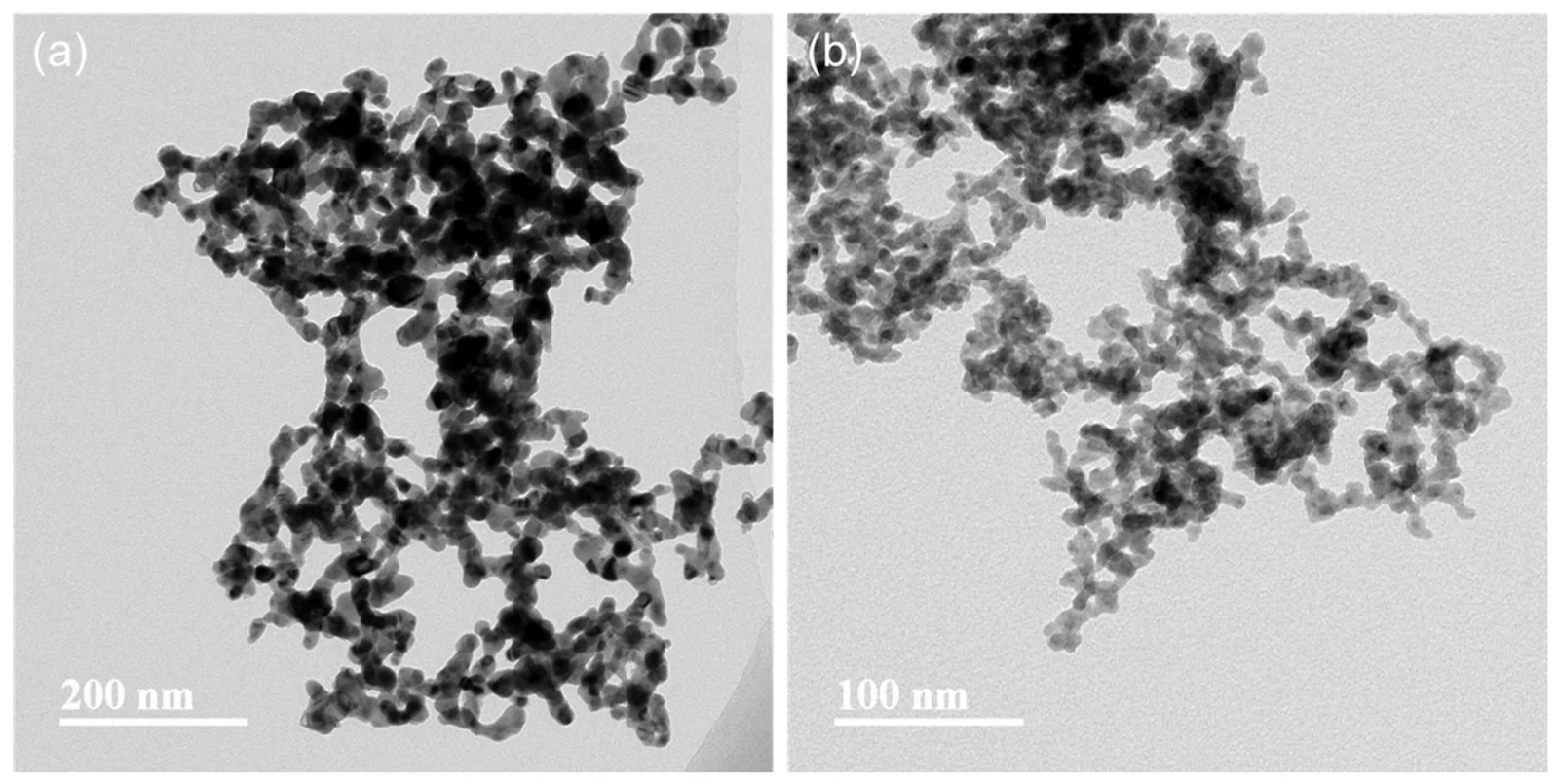
TEM images of (**a**) Cu aerogel and (**b**) CuZn alloy aerogel.

Cu and CuZn aerogels were prepared by our previously reported one-step method [27,28]. Transmission electron microscopy (TEM) was first employed to visualize the microstructural features of as-prepared pure Cu aerogel and CuZn alloy aerogel. As shown in Figure 1, both samples consist of interconnected spherical nanoparticles cross-linking into self-supporting three-dimensional porous network architectures. The voids formed between interconnected nanoparticles provide abundant sites for bacterial adhesion and diffusion channels for the sustained release of metal ions, which is beneficial to antibacterial application. Significant differences in particle size and dispersion state can be identified between the two samples. The pure Cu aerogel (Figure 1a) possesses larger primary nanoparticles, and severe particle aggregation can be clearly observed. Numerous Cu nanoparticles stack tightly together, which reduces the number of exposed active sites. In contrast, the CuZn alloy aerogel (Figure 1b) exhibits obviously refined nanoparticles with a narrower particle size distribution. The introduction of Zn effectively alleviates particle agglomeration and promotes uniform dispersion of nanoparticles within the aerogel network.

**Figure 2:**
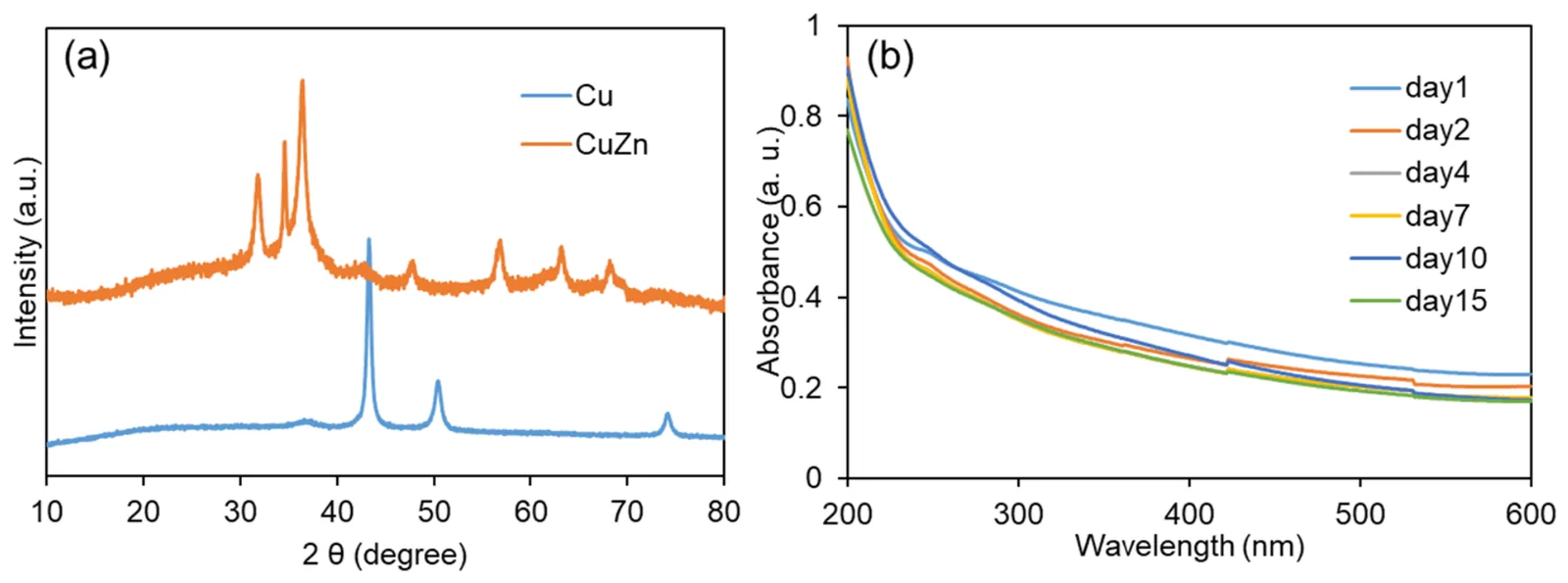
(**a**) XRD spectrum of Cu and CuZn. (**b**) Time-dependent UV–vis absorption spectra of CuZn alloy aerogel aqueous suspension.

X-ray diffraction (XRD) measurements were then performed to identify the crystalline phase composition of the as-prepared pure Cu aerogel and CuZn alloy aerogel, and the corresponding patterns are displayed in Figure 2a. For the single-component Cu aerogel (blue line), three distinct diffraction peaks located at 2θ = 43.3°, 50.4° and 74.1° were assigned to the (111), (200) and (220) crystal planes of metallic copper (JCPDS card No. 04-0836) [29]. No diffraction signals assigned to copper oxides were detected, demonstrating that the pure Cu aerogel consisted solely of crystalline Cu without oxidation impurities. After introducing Zn, obvious shifts and variations in diffraction peaks were observed. All characteristic peaks originating from Cu shifted toward lower 2θ values. According to Bragg’s law, the decreased diffraction angle indicates an enlarged interplanar spacing, which arises from lattice tensile distortion induced by the substitution of larger Zn atoms into the Cu lattice, indicating the probable formation of a Cu-Zn substitutional solid solution.

Inductively coupled plasma optical emission spectrometry (ICP-OES) was employed to quantify the metal elemental composition of CuZn alloy aerogel. The measured molar ratio of Cu to Zn in the obtained product was approximately 2.79:1, which obviously deviated from the initial feeding molar ratio of 1:1 during preparation. This phenomenon might be attributed to the different complexation and deposition behaviors of the two metal ions throughout the sol–gel process. Cu^2+^ tends to interact preferentially with the gel matrix and is immobilized within the three-dimensional network. In contrast, Zn^2+^ exhibits weaker binding affinity toward the gel framework, and partial Zn^2+^ is easily lost during the washing procedure, resulting in Cu enrichment in the final aerogel. Even so, ICP results confirm that both Cu and Zn are successfully incorporated into the aerogel skeleton, which facilitates the formation of alloy structure. It should be noted that the present work focuses on investigating this specific composition, and we do not intend to claim that the obtained Cu:Zn molar ratio of 2.79:1 represents the optimal antibacterial composition.

To evaluate the long-term photooxidation resistance of CuZn alloy aerogel in aqueous environments, we prepared an aqueous suspension of the material and recorded its UV–vis absorption spectra over 15 days, as shown in Figure 2b. The material exhibits broad absorption across the 200–600 nm range. The absorbance only slightly decreased within 15 days, indicating that CuZn alloy aerogel has favorable anti-photoaging capacity in water and is suitable for long-term antibacterial applications in aqueous scenarios.

**Figure 3:**
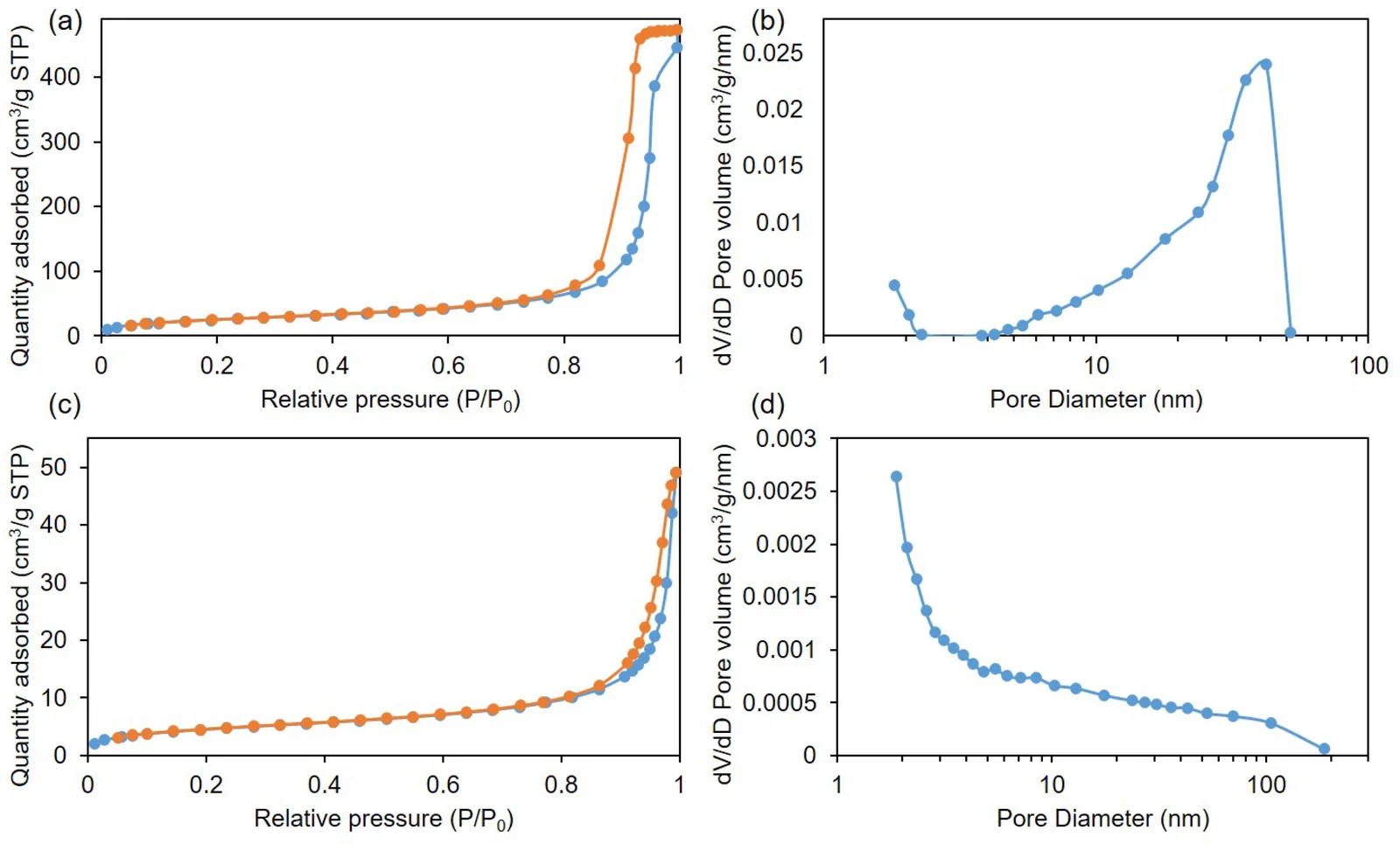
Nitrogen adsorption-desorption isotherms and pore-size distribution curves. (**a**) N_2_ adsorption-desorption isotherms of CuZn alloy aerogel; (**b**) pore-size distribution of CuZn alloy aerogel; (**c**) N_2_ adsorption-desorption isotherms of pure Cu aerogel; (**d**) pore-size distribution of pure Cu aerogel. For each isotherm plot (**a**,**c**), the lower branch denotes the adsorption process and the upper branch denotes the desorption process.

Figure 3 depicts the nitrogen adsorption–desorption isotherms and pore-size distribution profiles of CuZn alloy aerogel and Cu aerogel. As shown in Figure 3a,c, both samples exhibit Type-IV isotherms accompanied by distinct hysteresis loops, characteristic of mesoporous structures, where monolayer–multilayer adsorption and capillary condensation occur within mesoporous voids. The hysteresis loops belong to the H3 type, indicating slit-shaped pores and irregular voids originating from particle stacking, with relatively heterogeneous pore-size distributions and no obvious saturation plateau at high relative pressure. The CuZn alloy aerogel delivers much higher adsorbed volume and larger hysteresis-loop area compared with Cu aerogel, demonstrating its more developed porous architecture. The BET specific surface area and total pore volume of CuZn alloy aerogel are 91.21 m^2^/g and 0.731 cm^3^/g, which are approximately 5.5-fold and 9.6-fold higher than those of Cu aerogel (16.71 m^2^/g and 0.076 cm^3^/g), respectively. The pore-size distribution further reveals abundant mesopores centered at 20–30 nm for CuZn alloy aerogel, whereas Cu aerogel is dominated by small-size pores with limited mesopore fraction. These results confirm that Zn incorporation constructs a well-developed and interconnected mesoporous network. Such favorable porous features offer more accessible sites for bacterial contact, which serves as one contributing factor to the improved antibacterial performance of CuZn alloy aerogel.

### 2.2. Antibacterial Activity of Cu and CuZn Aerogels

To verify whether Zn alloying can improve the antibacterial capacity of monocopper aerogel as we hypothesized, concentration-dependent bacterial viability assays and agar diffusion tests were conducted.

**Figure 4:**
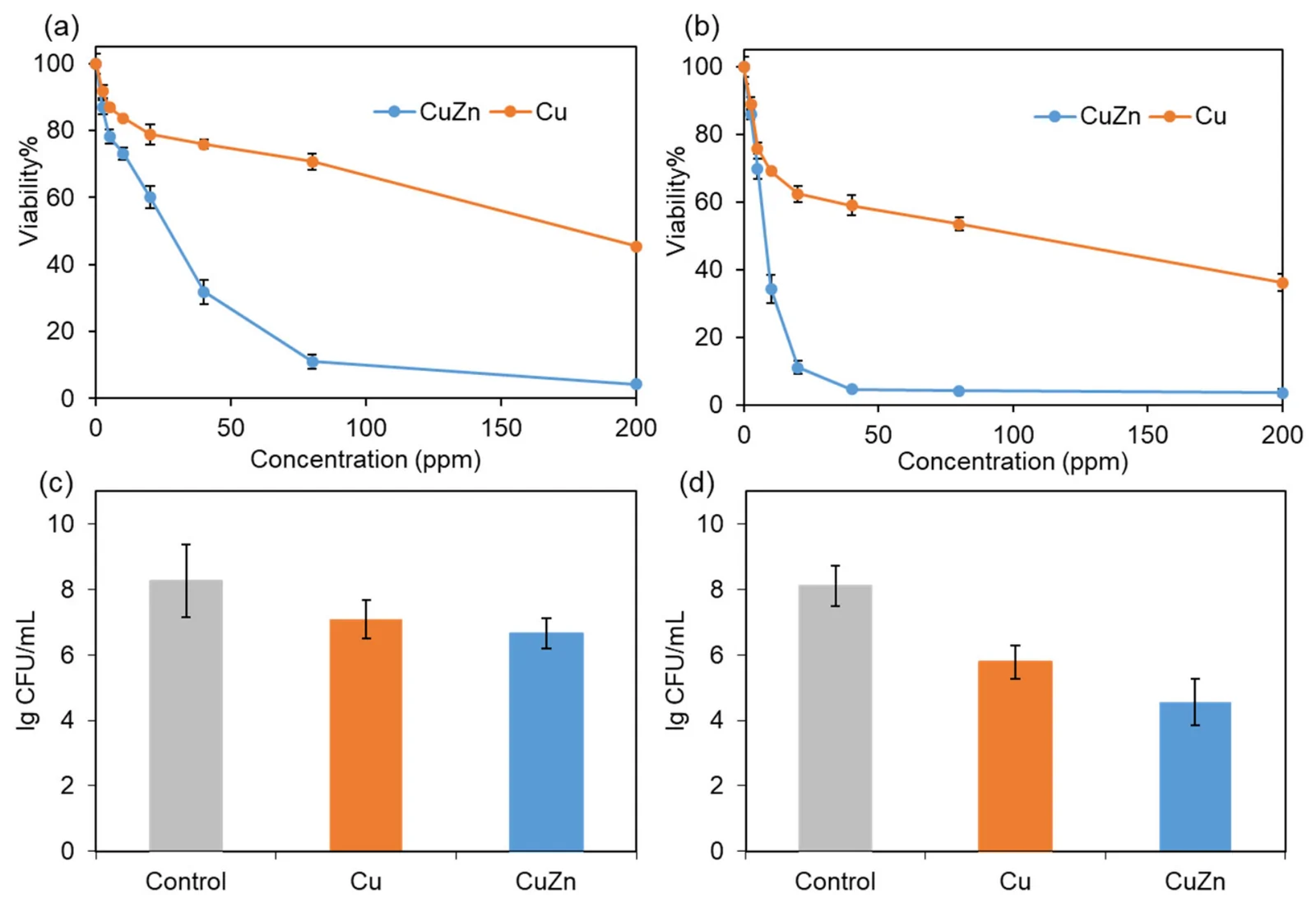
Antibacterial effect of Cu and CuZn on (**a**) *E. coli* and (**b**) *S. aureus*. Viable bacterial counts (lg CFU/mL) for (**c**) *E. coli* and (**d**) *S. aureus* determined by plate-counting assay at 40 ppm sample concentration. Data are expressed as mean ± SD.

First, to quantitatively evaluate the antibacterial activity of pure Cu aerogel and CuZn alloy aerogel, continuous concentration gradients ranging from 0 to 200 ppm were designed to record the bacterial viability curves against Gram-negative *E. coli* (Ec) and Gram-positive *S. aureus* (Sa). As illustrated in Figure 4a,b, bacterial viability decreased gradually with the elevation of material concentration. Within the tested concentration range, CuZn alloy aerogel exhibited remarkably stronger inhibitory effects against both strains than Cu aerogel. Taking *S. aureus* as an example, nearly complete growth inhibition was achieved at a CuZn alloy concentration of 40 ppm, whereas over 50% of bacterial cells remained viable in the Cu aerogel group at the same concentration. The distinctly lower bacterial viability observed for CuZn alloy aerogel at identical concentrations confirms the superior inhibitory effect originating from bimetallic Cu and Zn components.

It should be noted that the turbidity-based OD_600_ assay mainly reflects bacterial proliferation inhibition and cannot strictly distinguish bacteriostatic from bactericidal effects. Therefore, plate-counting CFU assays were further conducted at the representative concentration of 40 ppm to directly quantify viable cultivable bacteria. As shown in Figure 4c,d, Cu aerogel induced only a moderate reduction in colony counts, whereas CuZn alloy aerogel caused a substantial decrease in lg CFU/mL for both *E. coli* and *S. aureus*. These CFU results provide direct evidence that CuZn alloy aerogel possesses superior bactericidal activity. Combining the turbidity and CFU data, we conclude that CuZn alloy aerogel can not only suppress bacterial proliferation but also effectively kill viable bacteria.

**Figure 5:**
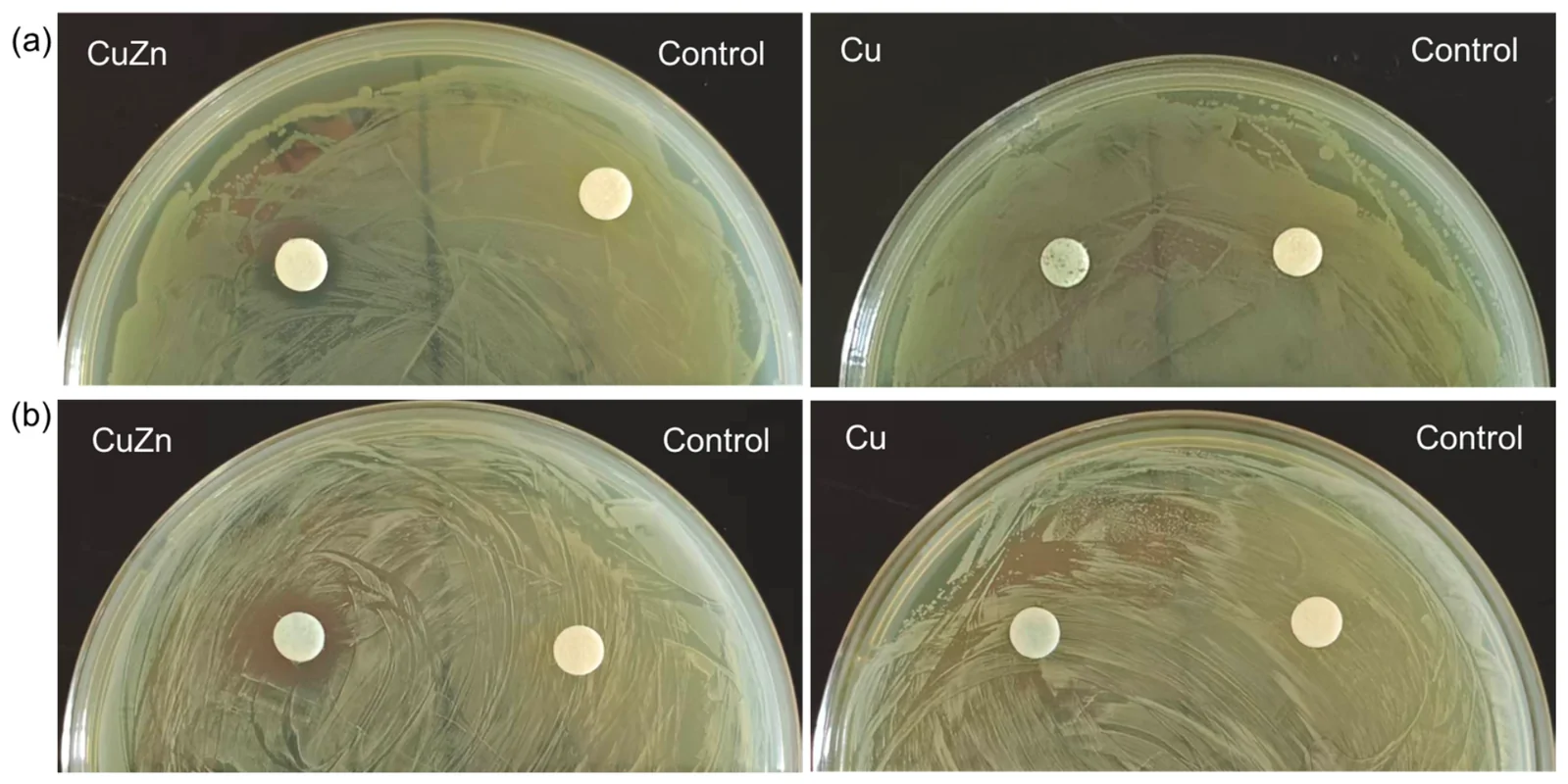
Antibacterial ability of Cu and CuZn tested using solid agar diffusion method. Representative results for (**a**) *E. coli* and (**b**) *S. aureus*.

We also adopted the disk diffusion method to visually compare the antibacterial performance of Cu aerogel and CuZn alloy aerogel. All samples were prepared at a unified concentration of 0.5 mg/mL, and plate antibacterial assays were carried out against *E. coli* and *S. aureus*, with blank filter paper serving as the control group. As shown in Figure 5, no transparent inhibition zone was observed around the blank filter paper. Only faint, indistinct inhibition zones were detected for the Cu aerogel group. Encouragingly, prominent clear inhibition zones formed for the CuZn alloy aerogel treatment group, which further verifies that CuZn alloy aerogel possesses superior antibacterial capacity compared with Cu aerogel.

**Figure 6:**
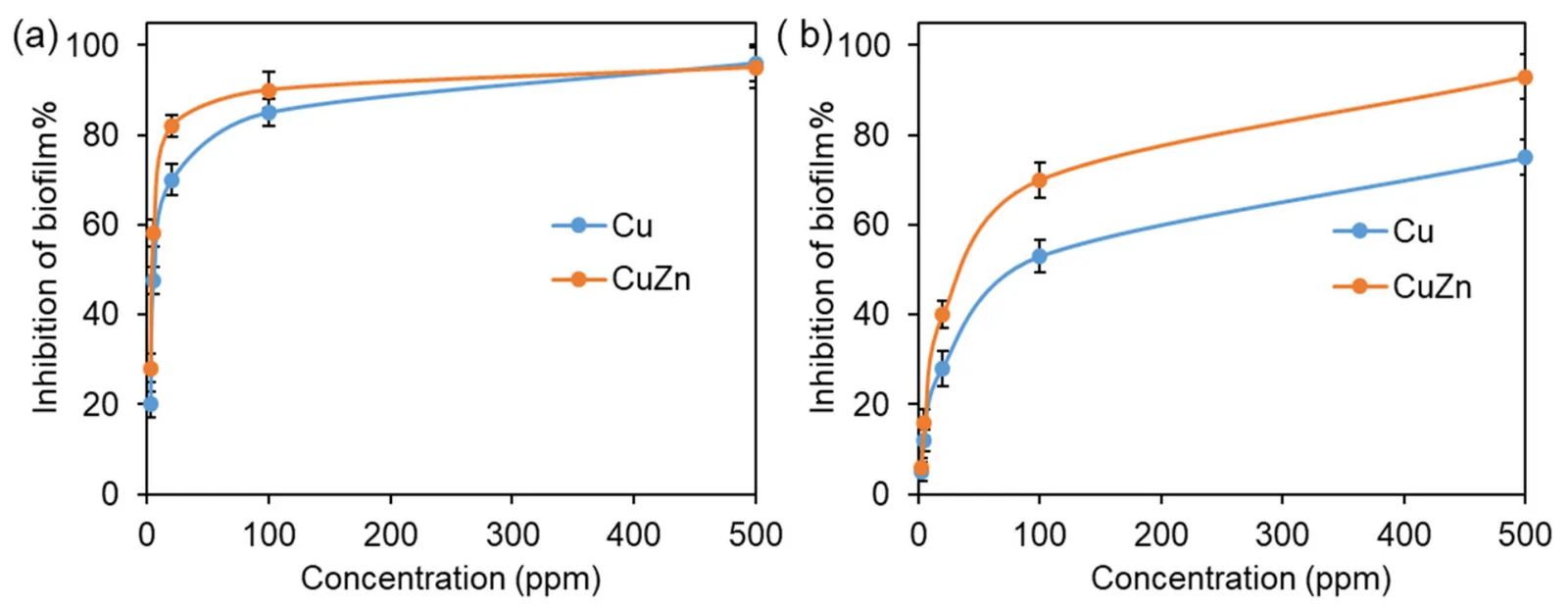
Percentage of biofilm inhibition of Cu and CuZn against (**a**) *E. coli* and (**b**) *S. aureus*. Data are expressed as mean ± SD.

Bacterial biofilms are critical contributors to persistent infections and elevated bacterial drug resistance. Quantitative measurements of biofilm biomass of *E. coli* and *S. aureus* were further performed to compare the anti-biofilm capacity of Cu aerogel and CuZn alloy aerogel. As shown in Figure 6, both materials exerted concentration-dependent inhibitory effects on biofilm formation of the two strains, and the biofilm inhibition rate increased continuously with the rising material concentration. For *E. coli*, the discrepancy in biofilm inhibition efficiency between Cu and CuZn was insignificant, and comparable inhibition rates were achieved at 500 ppm. In the case of *S. aureus*, a prominent gap in anti-biofilm performance was observed: CuZn alloy aerogel achieved a biofilm inhibition rate of 93% at 500 ppm, while the inhibition rate of Cu aerogel only reached approximately 75%. These results verify that the Cu–Zn bimetallic system can not only efficiently eliminate planktonic bacteria but also restrain biofilm formation. Combined with the foregoing data of planktonic bacterial viability and disk diffusion assays, it is collectively demonstrated that CuZn alloy aerogel integrates potent bactericidal activity and excellent anti-biofilm performance, exhibiting far superior comprehensive antibacterial properties relative to Cu aerogel.

### 2.3. Antibacterial Mechanisms of Cu and CuZn Aerogels

**Figure 7:**
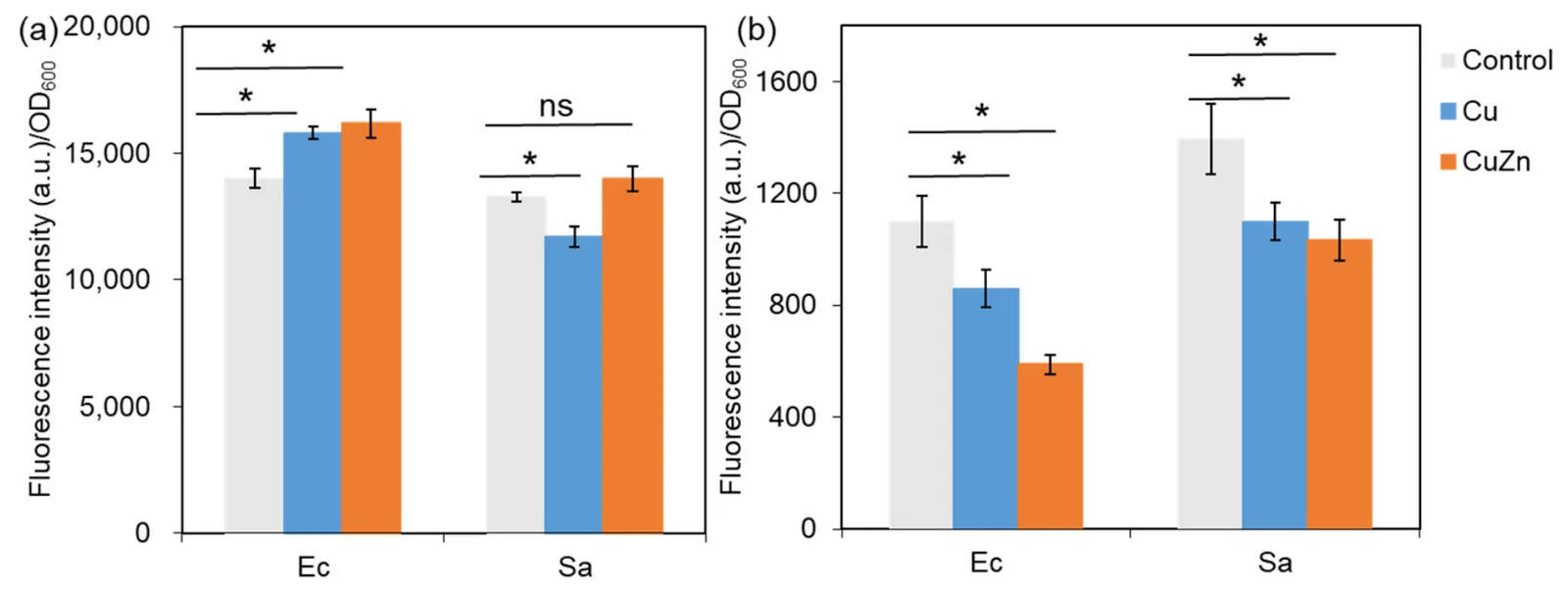
(**a**) Changes in intracellular ROS levels and (**b**) bacterial membrane potential changes after Cu and CuZn treatment. The asterisks (*) indicate significant differences between the groups (*p* < 0.05), and ‘‘ns’’ indicates no significance between the groups. Data are expressed as mean ± SD.

To elucidate the underlying antibacterial mechanism, intracellular reactive oxygen species (ROS) levels and bacterial membrane potential were further measured. Excessive intracellular ROS trigger bacterial oxidative stress and cause irreversible oxidative damage to DNA, proteins and enzyme systems, which acts as the core bactericidal pathway of metal-based antibacterial materials. Fluorescence staining quantification was performed to detect the intracellular ROS fluorescence intensity of *E. coli* and *S. aureus* treated with blank control, Cu aerogel and CuZn alloy aerogel. As illustrated in Figure 7a, after treatment with the two aerogels, the intracellular ROS fluorescence intensity of *E. coli* was elevated in both material groups, and slightly higher ROS accumulation was observed in the CuZn alloy group. In contrast, distinct trends were found for *S. aureus*. Compared with the blank group, the intracellular ROS level decreased upon Cu aerogel treatment. Although CuZn alloy aerogel slightly increased ROS production, the growth range was limited. These results reveal that the capacity of the CuZn alloy to trigger bacterial oxidative stress varies by strain; it effectively boosts ROS generation against *E. coli*, yet exhibits weak ROS-inducing activity toward *S. aureus*.

Membrane potential is a critical physiological indicator that sustains bacterial energy metabolism and transmembrane substance transport. Imbalance of membrane potential directly triggers membrane rupture and leakage of intracellular components, ultimately resulting in bacterial inactivation. The fluorescence intensity of membrane potential for bacteria in each group is presented in Figure 7b. The blank control group exhibited the highest membrane potential fluorescence intensity for both strains, while remarkable declines in fluorescence intensity were detected in both Cu aerogel and CuZn alloy aerogel treatment groups. For *E. coli*, the CuZn alloy aerogel displayed the minimum fluorescence intensity, corresponding to the most severe membrane damage. In the *S. aureus* system, the fluorescence intensity of the CuZn aerogel was also lower than that of the Cu aerogel group. Collectively, these findings demonstrate that the Cu–Zn bimetallic system exerts more destructive effects on bacterial cell membranes. These mechanistic results explain why CuZn alloy aerogel achieves superior antibacterial performance compared with single Cu aerogel, which matches our initial assumption that the Cu–Zn bimetallic combination may bring combined bactericidal effects.

Notably, CuZn alloy aerogel triggered only a modest elevation of intracellular ROS for *S. aureus*, yet it still exerted potent growth-inhibitory effects against this Gram-positive bacterium. This strain-dependent response can be interpreted from the perspective of bacterial cell-wall architecture. *S. aureus* possesses a thick, peptidoglycan-rich cell wall, which renders intracellular ROS overproduction not the dominant lethal pathway for this species [30]. Instead, severe membrane-potential depolarization constitutes the major inhibitory mechanism; membrane depolarization disturbs essential energy metabolism, increases cytoplasmic membrane permeability, and causes leakage of intracellular biomolecules, ultimately suppressing bacterial viability. For Gram-negative *E. coli*, by contrast, both ROS-mediated oxidative stress and membrane-potential disruption jointly contribute to antibacterial activity. These observations highlight that bimetallic metal aerogels can operate via distinct dominant inhibitory routes depending on bacterial strain.

### 2.4. Cytotoxicity of Cu and CuZn Aerogels

**Figure 8:**
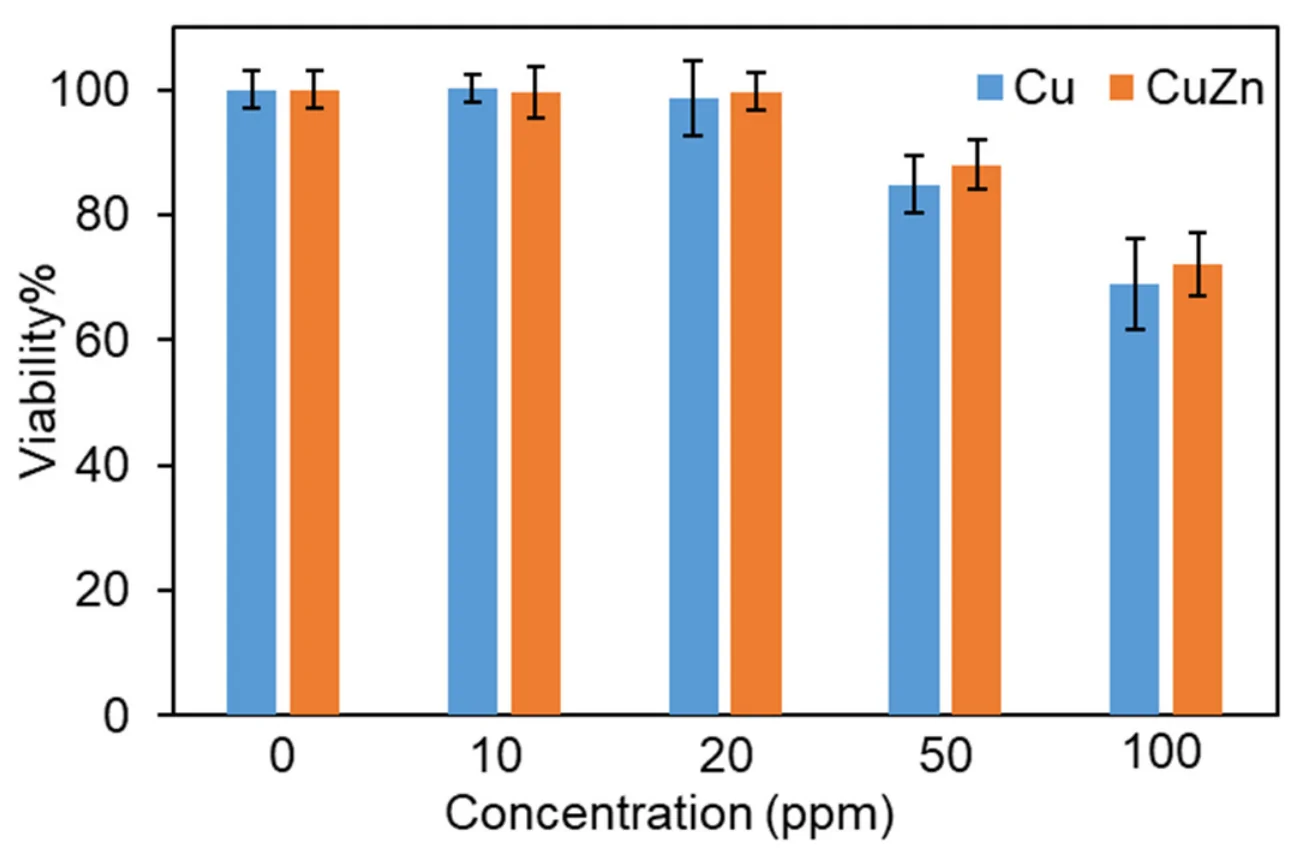
Cell viability of DC2.4 cells treated with Cu aerogel and CuZn alloy aerogel for 24 h. Data are expressed as mean ± SD.

To evaluate the cytocompatibility of Cu aerogel and CuZn alloy aerogel, dendritic DC2.4 cells were utilized to perform cell viability assays with a concentration gradient ranging from 0 to 100 ppm, and the cell viability results are illustrated in Figure 8. At concentrations of 50 ppm and below, the viability of DC2.4 cells incubated with both materials remained higher than 85%, demonstrating negligible cytotoxicity and satisfactory biosafety within this dosage range. When the concentration was increased to 100 ppm, the cell viability of the CuZn alloy aerogel group was still maintained above 70%. Combined with the antibacterial data described above, CuZn alloy aerogel exhibits remarkable bactericidal and anti-biofilm activities at 100 ppm, at which concentration DC2.4 cell viability remains above 70%. These observations indicate a favorable preliminary balance between antibacterial potency and cellular biosafety under the tested conditions.

## 3. Conclusions

In this work, guided by the inherent antibacterial capacity of Cu nanoparticles and low-toxicity Zn metal, we synthesized monometallic Cu aerogel and CuZn alloy aerogel via a one-step strategy to explore the bimetallic effect on antibacterial performance. Structural characterizations confirmed the probable formation of Cu–Zn substitutional solid solution, where Zn effectively refined nanoparticles and mitigated aggregation. Systematic biological tests demonstrated that CuZn alloy aerogel achieved remarkably enhanced broad-spectrum bactericidal and anti-biofilm effects against *E. coli* and *S. aureus* compared with pure Cu aerogel. Mechanism studies uncovered two important antibacterial pathways, including strain-dependent intracellular ROS accumulation and severe disruption of bacterial membrane potential. Moreover, DC2.4 cell viability assays verified its satisfactory cytocompatibility, as cell viability remained above 70% at 100 ppm with robust antibacterial activity. Overall, this study demonstrates the superior antibacterial performance of Cu-Zn alloy aerogel, which arises from a combination of refined particle size, well-developed porous architecture, and potential bimetallic-alloy-related chemical effects. This work provides a simple route to prepare biocompatible metal aerogels that are promising for biomedical antibacterial scenarios. Further systematic investigations including component-gradient optimization, physical-mixture control experiments, quantitative synergy evaluation, ion-release kinetics and advanced alloy characterizations are worthy of exploration in future work.

## 4. Materials and Methods

### 4.1. Preparation of Cu and CuZn Aerogels

For the synthesis of CuZn aerogel, NaBH_4_ (58 mg) was dissolved in 5 mL water and added quickly to an aqueous solution (145 mL) containing Zn(NO_3_)_2_ (0.25 mM) and Cu(NO_3_)_2_ (0.25 mM). The mixture was then placed in a water bath (45 °C) and incubated for 6 h. Then black precipitate was collected and washed five times with deionized water. The final product (black powder) was obtained by freeze-drying.

For the synthesis of Cu aerogel, the concentration of Cu(NO_3_)_2_ was set to 0.5 mM, while all other dosages and experimental procedures remained consistent.

In this one-step sol–gel procedure, metal ions are reduced in situ by NaBH_4_ to generate metallic nanoparticles. Freshly formed nanoparticles possess high surface energy and spontaneously assemble and cross-link into continuous three-dimensional gel networks within the same reaction system without additional intermediate isolation or post-gelation treatment. Subsequent washing removes residual NaBH_4_ and by-products, and freeze-drying preserves the pre-formed interconnected gel network to yield the final aerogel powder. Therefore, the network-forming gelation process is accomplished in a single reaction vessel, which justifies the “one-step sol–gel” denomination, whereas washing and freeze-drying are only post-processing steps.

### 4.2. Characterization of Cu and CuZn Aerogels

A transmission electron microscope (TEM, TECNAI G2 Spirit TWIN, Hillsboro, OR, USA), X-ray diffractometer (XRD, SmartLab-SE, Tokyo, Japan) and Inductively Coupled Plasma Optical Emission Spectrometer (ICP-OES, Agilent 720ES, Santa Clara, CA, USA) were used to characterize the morphology, crystalline structure and elemental composition of the two aerogels. A UV-Vis spectrophotometer (Persee, TU-1901, Beijing, China) was used to test the stability of CuZn aerogel in water.

### 4.3. Bacterial Culture

*E. coli* and *S. aureus* were used for antibacterial experiments. Both bacteria were cultured in Liquid Luria–Bertani (LB) medium (5 g yeast extract, 10 g tryptone and 10 g NaCl in 1 L water).

### 4.4. Growth Inhibition Assay

The overnight-cultured bacteria were first diluted in LB medium to an OD_600_ value (optical density at 600 nm) of 0.02. Then, 50 μL of the diluted suspension was transferred to a 96-well plate, followed by addition of Cu and CuZn aerogels with different concentrations, and the final volume was 100 μL. The 96-well plate was then cultured at 37 °C for 24 h. OD_600_ was then tested using a microplate reader, and the calculation formula for bacterial viability is expressed as (OD_600_ value of aerogel-treated group/OD_600_ value of untreated blank group) × 100%.

### 4.5. Antibacterial Zone Test

The bacterial inhibition effect of 0.5 mg of Cu and CuZn aerogels was evaluated using the solid agar diffusion method in Mueller–Hinton plates [31]. Overnight-cultured *E. coli* and *S. aureus* suspensions were diluted with sterile normal saline to adjust the OD_600_ value to 0.01 (approximately 1.0 × 10^6^ CFU/mL). A volume of 100 μL bacterial suspension was evenly spread onto nutrient agar plates, followed by air-drying at room temperature for 3–5 min. Sterile tweezers were used to place prefabricated Cu aerogel and CuZn alloy aerogel separately on the agar surface. Each sample was gently pressed to ensure tight contact with the agar, with a spacing of more than 25 mm between different samples. Blank filter paper disks without any aerogel materials were set as the negative control. Finally, all plates were incubated at 37 °C for 24 h.

### 4.6. Biofilm Inhibition Assay

Overnight cultures of the two tested bacterial strains were diluted with fresh culture medium to an OD_600_ value of 0.01. A volume of 150 µL bacterial suspension was added into each well of a 96-well plate, followed by immediate addition of 100 µL serially diluted test samples to establish a series of preset final concentrations in each well. The 96-well plate was then incubated statically at 37 °C for 36 h to allow biofilm maturation. After incubation, planktonic bacterial suspensions were gently decanted, and each well was washed with deionized water 3 times. The plate was inverted onto absorbent filter paper and tapped lightly to thoroughly drain residual liquid. Subsequently, 0.1% crystal violet staining solution was added to each well, and staining was performed on an orbital shaker at 70 r/min for 30 min at room temperature. Upon completion of staining, the crystal violet solution in each well was discarded and washed with deionized water. Finally, 250 µL of 30% acetic acid solution was added. The plate was shaken gently to fully dissolve and homogenize the eluted crystal violet. The absorbance at 595 nm (OD_595_) was then tested by a microplate reader, and the absorbance value was adopted to quantitatively characterize the biomass of biofilm formed under each sample concentration [32].

### 4.7. Membrane Potential and ROS Detection

The method of membrane potential and ROS detection was the same as our previously reported method [33].

For membrane potential detection, 100 μL of bacteria suspension (OD_600_ = 0.5) treated with Cu and CuZn aerogels (50 ppm) was cultured in a 96-well plate at 37 °C for 2 h. Then 1 μM Rhodamine-123 was added and incubated for another 3 h. The cells were collected by centrifugation and resuspended in PBS. The fluorescence intensity at 520 nm was recorded by a microplate reader (emission wavelength = 520 nm).

For ROS detection, 100 μL of bacterial solution with an OD_600_ value of 0.5 was added to a 96-well plate, with the concentrations of Cu and CuZn aerogels being 50 ppm. After incubation at 37 °C for 2 h, 1 μM of 2′,7′-dichlorofluorescin diacetate (DCFH-DA) was added and incubated for 0.5 h. The fluorescence intensity at 520 nm was detected by a microplate reader (with an excitation wavelength of 488 nm).

### 4.8. Cell Viability Test

DC2.4 cells (purchased from Shanghai Boke Biotechnology Co., Ltd., Shanghai, China) were cultured in DMEM medium supplemented with 10% (*v*/*v*) fetal bovine serum, 100 U mL^−1^ penicillin, and 100 U mL^−1^ streptomycin. Cell incubation conditions were set at 37 °C with 5% CO_2_ in a humidified atmosphere. The cells were seeded in a 96-well plate and pre-cultured for 24 h. Then, Cu or CuZn aerogel with gradient concentrations (10, 20, 50 and 100 ppm) was added into the cell culture system for another 24 h of incubation. CCK-8 assay kits (Solarbio, Beijing, China) were utilized to measure the cell viability.

### 4.9. Statistical Analysis

All biological experiments were conducted in triplicate; experimental data were presented as mean value ± standard deviation (SD). Differences with *p* < 0.05 were regarded as statistically significant.

## Data Availability

Data may be available from the corresponding author, Shuo Liu, upon request.
